# Nanoencapsulation-Based Edible Coating of Essential Oils as a Novel Green Strategy Against Fungal Spoilage, Mycotoxin Contamination, and Quality Deterioration of Stored Fruits: An Overview

**DOI:** 10.3389/fmicb.2021.768414

**Published:** 2021-11-26

**Authors:** Somenath Das, Abhinanda Ghosh, Arpan Mukherjee

**Affiliations:** ^1^Department of Botany, Burdwan Raj College, Purba Bardhaman, India; ^2^Institute of Environment and Sustainable Development, Banaras Hindu University, Varanasi, India

**Keywords:** essential oil, nanoencapsulation, antifungal, antimycotoxigenic, preservative

## Abstract

Currently, applications of essential oils for protection of postharvest fruits against fungal infestation and mycotoxin contamination are of immense interest and research hot spot in view of their natural origin and possibly being an alternative to hazardous synthetic preservatives. However, the practical applications of essential oils in broad-scale industrial sectors have some limitations due to their volatility, less solubility, hydrophobic nature, and easy oxidation in environmental conditions. Implementation of nanotechnology for efficient incorporation of essential oils into polymeric matrices is an emerging and novel strategy to extend its applicability by controlled release and to overcome its major limitations. Moreover, different nano-engineered structures (nanoemulsion, suspension, colloidal dispersion, and nanoparticles) developed by applying a variety of nanoencapsulation processes improved essential oil efficacy along with targeted delivery, maintaining the characteristics of food ingredients. Nanoemulsion-based edible coating of essential oils in fruits poses an innovative green alternative against fungal infestation and mycotoxin contamination. Encapsulation-based coating of essential oils also improves antifungal, antimycotoxigenic, and antioxidant properties, a prerequisite for long-term enhancement of fruit shelf life. Furthermore, emulsion-based coating of essential oil is also efficient in the protection of physicochemical characteristics, *viz*., firmness, titrable acidity, pH, weight loss, respiration rate, and total phenolic contents, along with maintenance of organoleptic attributes and nutritional qualities of stored fruits. Based on this scenario, the present article deals with the advancement in nanoencapsulation-based edible coating of essential oil with efficient utilization as a novel safe green preservative and develops a green insight into sustainable protection of fruits against fungal- and mycotoxin-mediated quality deterioration.

## Introduction

In the busy lifestyle of the current generation, consumers have become increasingly focused on healthy foods, especially on fresh fruits because they are rich sources of micronutrients, minerals, and bioactive constituents, which provide maximum health benefits such as antioxidant, antidiabetic, anti-inflammatory, and anti-allergic activities (Prakash et al., 2018). Fruits are also regarded as protective foods, suggested to be incorporated into the human daily diet due to the presence of vitamins, proteins, and trace elements such as manganese, copper, and zinc, which actively participate in the metabolic functioning of the body (Jahan et al., 2011). During storage, the quality of fruits deteriorates, resulting in unexpected flavors, softening of the outer surface, browning, water loss, and breakdown of surface textures. Furthermore, storage conditions also facilitate the infestation of fungal flora and secretion of mycotoxin, which is a major problem especially affecting the fruits’ nutritional properties (Pessu et al., 2011). Consequently, mycotoxin secretion reinforces the production of reactive oxygen species, which could lead to lipid peroxidation (Kalagatur et al., 2018) and the ultimate shortening of the shelf life of fruits along with subsequent reduction in consumer acceptance. In addition to nutritional loss, postharvest fungal contamination may collapse the fruits’ market value by increasing transport and storage costs. Synthetic preservatives or chemical disinfectants are commonly employed to combat fungal spoilage and mycotoxin contamination of fruits. Disinfectants containing chlorine and hypochlorite are not very effective in reducing fungal proliferation; however, their excessive application may cause skin irritation, respiratory and gastrointestinal problems (Issa-Zacharia et al., 2010). Moreover, ozone, peroxyacetic acid, organic acid, and hydrogen sulfide are not able to achieve maximum inhibition and also possess potential side effects and toxicity (Ramos et al., 2013). Essential oils, a major group of higher plant secondary metabolites, allow for the retardation of fungal spoilage and mycotoxin contamination and eventually help in the replacement of synthetic antimicrobials (Das et al., 2021a). However, the practical application of essential oils as a fumigant has several limitations such as easy volatility, rapid biodegradability, less solubility, less stability, and negative effect on organoleptic properties (Barradas and de Holanda e Silva, 2021). Hence, there is a pressing need for control delivery of essential oil components, which can be processed through a novel nanoencapsulation technology. Nanoencapsulation simply demonstrates the entrapment of essential oils into a carrier matrix providing stability and also protection from external fluctuating environmental conditions, leading to improvement in biological efficacy (Das et al., 2021a). Proteins, polysaccharides, and lipids are the most commonly used encapsulating matrix for efficient imprisoning of essential oils, having the qualities of high water solubility, biodegradability, and easy availability, which are obtained from plants (starch, cellulose, and gluten), animals (dextrin, chitosan, and casein), and marine and microbial sources (Martín et al., 2010; Vishwakarma et al., 2016). Various encapsulation techniques such as ionic gelation, coacervation, liposome, nanoemulsion, nanoprecipitation, and drying processes are commonly employed for encapsulation of essential oil and bioactive compounds to enhance their bioefficacy (Ezhilarasi et al., 2013). Currently, modern food scientists have suggested the implementation of nanoemulsion-based edible coating of essential oils around various stored food products for controlling food physicochemical characteristics such as respiration, firmness, color, and fungal infestation (Hasan and Nicolai, 2014). Edible coating constitutes a thin layer after dipping the food materials into an essential oil-based emulsion system which provides an extra protection similar to modified atmospheric packaging (Maringgal et al., 2020). Nanoformulation-based edible coating opens a new window for application of essential oils in protection of fruits, especially during postharvest storage conditions. Among different synthesized formulations of essential oils, nanoemulsion is an innovative approach for enhancement in quality attributes of fruits by maintaining their flavor, color, and antioxidant properties (Hasan et al., 2020).

A very limited number of studies have been demonstrated for nanoemulsion-based coating of essential oils for protection against fungal and mycotoxin contamination and for maintenance of the physicochemical properties of fruits. On the basis of this consideration, the present review has focused on updated information regarding nanoformulation-based edible coating of essential oil on fruits to improve bioefficacy, particularly focusing on the mechanism of action, improvement in antioxidant activity, sensory attributes, and possibility for future industrial implementation as novel preservatives.

## Fungal and Mycotoxin Contamination of Stored Fruits

Fruits are recognized as a prime substrate for fungal spoilage in postharvest conditions. The minimally processed fruits are more prone to fungal proliferation due to different processing such as peeling, slicing, and cutting, which expose the surface and release some nutritive constituents, leading to maximum contamination (Rojas-Graü et al., 2007; Siroli et al., 2015). Species of *Aspergillus*, *Penicillium*, and *Alternaria* are major fungal flora infesting the surface of fruits and produce some host-specific mycotoxins causing effective biodeterioration of nutritional quality, which has added a new dimension of postharvest problem for fruits (Soliman et al., 2015). Occurrence of different fungal flora in fruits depends on the water activity; for example, when the water activity is higher than 0.9, there is more chances of *Fusarium* infestation, whereas, at maturity, the fruits become dehydrated with prevalent occurrence of Aspergilli (Magan and Aldred, 2007; Sanzani et al., 2016). In storage conditions, species of *Alternaria* and *Penicillium* are of great concern, causing maximum spoilage of apples by decay and mycotoxin contamination (Doores and Splittstoesser, 1983; Ostry et al., 2004). Patulin is a common mycotoxin in apples during storage produced by *Penicillium expansum*. Association of patulin was observed at the cracked surfaces of apples, and its limit often exceeded 50 ppb after postharvest managements (Ostry et al., 2004). Barkai-Golan and Paster (2008) reported *Aspergillus flavus* and *Aspergillus parasiticus* infestation in grape fruits along with production of ochratoxin A and aflatoxin, leading to the deterioration of organoleptic and physicochemical properties. Senyuva et al. (2007) described the occurrence of aflatoxins in dried figs (collected from Turkey) due to poor postharvest preservation processes. Ahmed and Robinson (1997) demonstrated rising contamination of dates collected from the United Arab Emirates by aflatoxin B_1_ and G_1_ during the storage periods. Higher relative humidity (66–93%) has been pointed out as a major factor for *A. parasiticus* infestation and aflatoxin contamination in the storage conditions. *Alternaria* is a commonly occurring storage fungal flora, basically contaminating stored fruits by production of altertoxin, alternuene, and tenuazonic acid (Scott, 2004). A number of alternaria toxins such as alternariol monomethyl ether, alternariol, and alternuene have been shown to contaminate oranges, apples, melons, and lemons. Scott and Kanhere (2001) reported the level of tenuazonic acid and alternariol in between 500 and 58,800 ng/g in apples. Delgado and Gómez-Cordovés (1998) and Lau et al. (2003) presented the contamination of alternariol up to 6 ng/g in raspberry, grapes, and apple juice due to the existence of alternariol during improper postharvest storage practices. Elamin and Sakuda (2021) reported the contamination of *Ziziphus jujuba* var *spinosa* fruit by *A. flavus* and AFB_1_ in storage, especially during the ripening stage. Infestation of *Fusarium proliferatum* on banana fruits leading to excessive contamination with fumonisin B_1_ and deterioration of fruit quality (due to the decreasing effect of chitinase, β-1,3-glucanase, and phenylalanine ammonia lyase) has been recently demonstrated by Xie et al. (2021). Li et al. (2020) reported the occurrence and co-occurrence of alternariol, altenuene, tenuazonic acid, and tentoxin in rotten apples. Moreover, the synergistic, antagonistic, and additive effects of different mycotoxins determine the severity of quality deterioration in apples. Considerable information has been reported for ochratoxin A contamination in grape fruits. Higher moisture content during postharvest preservation influences the infestation of *Aspergillus carbonarius* with resultant production of ochratoxin A (Wagacha and Muthomi, 2008). Wang et al. (2018) reported the contamination of different mycotoxins such as ochratoxins, aflatoxins, alternariol, tentoxin, patulin, zearalenone, T2 toxin, and trichothecenes from 10 kinds of dried fruits and nuts (chestnut, hazelnuts, almonds, figs, pine nuts, jujubes, longans, raisins, walnuts, and persimmons) with a contamination frequency of 124/253 (level of quantification < 473.16 μg/kg). Occurrences of multi-mycotoxins, *viz.*, ochratoxins, aflatoxins, patulin, trichothecenes, and alternaria toxins, in 104 different fruit samples (21 pears, 28 melons, 30 grapes, and 25 jujubes) collected from Xinjiang regions of China have been investigated, and the level of contamination was determined through ultra-performance liquid chromatography coupled to ion mobility quadrupole time-of-flight mass spectrometry with a level of detection and level of quantification at 0.06–2.22 and 0.2–7.39 μg/kg, respectively (Fan et al., 2022).

## Factors Affecting Fungal Spoilage and Mycotoxin Secretion in Fruits

Fungal colonization and mycotoxin production depend on the variety of intrinsic and extrinsic factors in fruits. Intrinsic factors mainly include the initial factors contributing to contamination in postharvest conditions such as pH, water activity, nutritional status, and texture of the fruits. However, relative humidity, storage temperature, and atmospheric compositions are recognized as major extrinsic factors responsible for fungal spoilage and mycotoxin contamination.

Growth of storage fungi is markedly influenced by water activity (a_w_), which correlates its interaction with substrates. An investigation of Romero et al. (2007) described the maximum growth of *A. carbonarius* at a_w_ 0.95 and produced ochratoxin. Bellı et al. (2004) reported the highest growth rate of 9.11 mm/day at 0.98 a_w_ and the slowest growth rate at 0.88 a_w_ for *Aspergillus* section Nigri. Mitchell et al. (2004) also agreed with between a_w_ 0.95–0.99 maximum contamination of *A. carbonarius* in grapes. Esteban et al. (2006) demonstrated the highest ochratoxin production by *Aspergillus ochraceous* at 0.96–0.99 a_w_. More importantly, the *in vitro* studies illustrated the variation in a_w_ levels at initial and later stages in different growth media such as Yeast Extract Agar (YEA) and Czapek yeast extract agar (CYA) with variation in ochratoxin production. Mannaa and Kim (2017) reported infestation of three different types of fungal species based on the a_w_ level during initial, middle, and last phases of storage. Hydrophilic fungal species were firstly dominated followed by mesophilic and xerophilic fungal species. The optimum a_w_ for growth and proliferation of xerophiles, mesophiles, and hydrophiles has been recognized as 0.95, 0.95–1.00, and 1.00, respectively.

Low pH of fruits during storage particularly facilitates the infestation of molds and yeasts, causing maximum spoilage, because most of the bacteria are eliminated as they prefer a near-neutral pH (Zhao et al., 2020).

Temperature plays an important role in fungal growth and mycotoxin production during storage conditions. In a study of Hill et al. (1983), maximum contamination of *A. flavus* by growth and sporulation at 35°C has been demonstrated, while aflatoxin production occurred even at 33°C. Klich et al. (2009) suggested the variation in aflatoxin level between 24 and 30°C depending on the substrate and potentiality of the strain. During storage, high moisture content also facilitates the production of fumonisin B_1_ by different species of *Fusarium*, and the optimum temperature for synthesis of this mycotoxin was reported as 15–30°C. In addition to water activity, pH, temperature, solute concentration, time, atmospheric conditions, inoculum density, and potential are major factors for maximum contamination along with the production of mycotoxins. More importantly, mycotoxins are synthesized by the toxigenic fungal species as a response to environmental stress conditions (da Cruz Cabral et al., 2013). Recent investigation suggested optimum growth conditions, storage temperatures, and substrate properties as prime factors for excessive proliferation and sporulation of fungal species in fruits (Marín et al., 2021). Hussein et al. (2020) reported the ability of *A. flavus*, *A. niger*, and *Penicillium citrinum* for production of pectinase enzyme, leading to quality deterioration of strawberries. The highest level of pectinase activity was reported at pH 8.0, while at more acidity and alkalinity, pectinase production was considerably reduced. Osmotic pressure of the substrate is another important factor responsible for fungal proliferation and mycotoxin secretion (Daou et al., 2021). Most notably, foods (especially fruits) containing maximum sugar provide a suitable podium for fungal invasion with the ability to hydrolyze it and support the metabolic activities (Hamad et al., 2014). Liu et al. (2016) illustrated that an increment in concentration of soluble sugars such as maltose, sucrose, and glucose from 3.0 to 6.0% promoted AFB_1_ production in foods.

## Essential Oil for Preservation Purposes

### Properties

A number of synthetic fungicides have been employed for inhibition of fungal spoilage and mycotoxin secretion in fruits. However, the emergence of resistant fungal races, limitations of government approval for continuously effective fungicides, and huge public concern regarding the hazardous effects of synthetic fungicides on human health and environmental non-sustainability have increased the attention for their sufficient replacement with non-chemical methods. Essential oils are a complex mixture of 20–60 different phenolic and terpenoid components and isolated from a number of aromatic plants (Basak and Guha, 2018). They are critically synthesized in plants as defense regulators and can also exert antibacterial, antifungal, antimutagenic, immunomodulatory, anti-inflammatory, and antioxidant activities (Bakkali et al., 2008). Essential oils have been used in agriculture, health, food, and cosmetic industries with widescale acceptability. The composition of essential oils widely differs among different plant parts, phonological stages, climatic factors, edaphic factors, seasons, light intensity, photoperiods, and extraction methods (Mazzarrino et al., 2015). *In vitro* studies pointed out that terpene as a single component is less efficient for fungal inhibition, while terpenoids are active against a number of food-contaminating microorganisms (Hyldgaard et al., 2012).

### Fabrication of Essential Oil-Based Nanoformulation and Their Physicochemical Characterization

Essential oils are highly volatile, less water soluble, and easily oxidized in direct environmental conditions; therefore, it is hardly possible to maintain the fruit quality by inhibition of fungal infestation and mycotoxin contamination and their nutritional values during practical application in fruits. Moreover, the direct interaction of essential oils with fruit surfaces may cause alteration in the sensory properties of fruit, which could lead to consumer unacceptability in commercial markets (Prakash et al., 2018). In this context, nanoencapsulation of essential oil into biodegradable and biocompatible polymers strengthens the practical efficacy and helps in the controlled release of essential oil, which will lead to the enhancement of the shelf life of fruits (Mohammadi et al., 2015; Ansarifar and Moradinezhad, 2021). Different strategies have been applied regarding nanoencapsulation of essential oils in food industries, such as nanoemulsion, nanogels, nanocapsules, and nanoparticles with subcellular-size particles (de Matos et al., 2019). A number of proteins and carbohydrate polymers are capable of encapsulating essential oil through a cross-linking interaction and nanostructural compaction (Das et al., 2021b). However, the utmost criteria for the selection of biopolymer as an encapsulating agent are its inclusion under the Generally Recognized As Safe (GRAS) category with a biodegradable and environmentally friendly nature (Adeyeye et al., 2019). Three different types of biopolymers, *viz.*, polypeptides, polynucleotide, and polysaccharides, are broadly used with paramount significance to develop a variety of encapsulated materials (Rehman et al., 2020). Among different biopolymers, polysaccharides, especially chitosan, alginate, starch, and pectin, are recognized as excellent bioadhesive polymers and capable of binding with essential oils and have controlled- as well as targeted-release property (Martǎu et al., 2019).

Majority of the studies represent nanoemulsion as a suitable strategy for coating of fruits and enhancing their nutritional qualities (Al-Tayyar et al., 2020). Nanoemulsion is developed by two immiscible liquids by incorporation of the dispersion phase of essential oils into dispersed media of water especially synthesized as an oil-in-water emulsion (Espitia et al., 2019). The size of essential oil droplets in the emulsion ranges between 10 and 1,000 nm. Owing to small-size droplets, nanoemulsion can act as a suitable delivery vehicle with a targeted mechanism of action. Bioavailability of essential oil is increased by the emulsion system of encapsulation due to the greater surface-to-volume ratio and subcellular-size particles (Das et al., 2020b). Moreover, the essential oil-based nanoemulsion is a stable system due to increased gravitational separation and Brownian motion (Gharenaghadeh et al., 2017). The oil phase of nanoemulsion was prepared by dissolving essential oil into lipophilic components such as Tween-20, Tween-80, and dichloromethane. Two different methods, *viz.*, high energy and low energy, have been applied for synthesis of essential oil-loaded nanoemulsion. High-energy methods are constituted with intensive disruptive forces for breakdown of large particles into subcellular-size particles by high-pressure homogenizers, sonicators, and microfluidizers. Low-energy methods involve the spontaneous as well as phase inversion methods without involvement of high-speed homogenization by the help of an oil–water–emulsifier system (Sugumar et al., 2016a).

After synthesis of essential oil-loaded nanoemulsion, physicochemical characterizations of the prepared nanoemulsion are performed through dynamic light scattering (DLS), X-ray diffraction (XRD), Fourier transform infrared spectroscopy (FTIR), scanning electron microscopy (SEM), atomic force microscopy (AFM), differential scanning colorimetry (DSC), and thermogravimetric analysis (TGA). A DLS study simply measures the size of particles in nanoscale regimes (Das et al., 2021c). SEM analysis confirms the nanometric size of particles with a greater surface-to-volume ratio and smooth surface with somewhat agglomeration at some places (Hasheminejad et al., 2019). It has also been reported that the size of nanoparticles was higher in the DLS technique as compared to SEM, which has been associated with aggregation and swilling of particles during dispersion in water (Esmaeili and Asgari, 2015). XRD analysis confirms the destruction of crystallinity after incorporation of the essential oil into the matrix polymer, leading to the development of amorphous structures of nanoparticles (Hadidi et al., 2020). In order to understand the molecular interaction between the matrix polymer and essential oils, FTIR is considered as an important parameter. Changing peak intensity along with variation in wave number suggests proper entrapment of essential oil into the biopolymer matrix (Yilmaz et al., 2019). In addition to size and shapes, AFM is also indicative of surface morphology, showing three-dimensional structures of essential oil-loaded nanoparticles and nanocomposites (Anand et al., 2021). DSC is a common method for examining the thermal sensitivity/stability and formation of solid-state complexes after proper encompassment of essential oils into the biopolymer matrix (Hadidi et al., 2020). Mass loss of nanoparticles with a maximum degradation rate at different steps has been observed in TGA thermogram. Decomposition of nanoparticles/nanocomplexes/nanocomposites at higher temperatures as compared to their free forms indicated the thermal stability of essential oils after proper encapsulation (Hadidi et al., 2020). For instance, to measure the nanometric size of linalool-loaded chitosan nanoemulsion composite, a DLS-based Zeta sizer, SEM, and AFM have been used (Das et al., 2021a). Liu and Liu (2020) measured the stability of chitosan nanoemulsion loaded with thymol and thyme essential oil by TGA. Thermal stability has been pointed out as an important property of nanoemulsion for food preservation because of the direct relationship between temperature and manufacturer’s processing during practical applications. The characteristic thermogram peak in DSC also revealed proper entrapment of oregano essential oil into the chitosan biopolymer through the electrospraying method. Wu et al. (2016) presented the nano-range size of emulsion particles in edible coating of citrus essential oil-loaded nanoemulsion by SEM and TEM analyses.

### Procedure for Edible Coating of Essential Oil Nanoemulsion in Fruits

**FIGURE 1:**
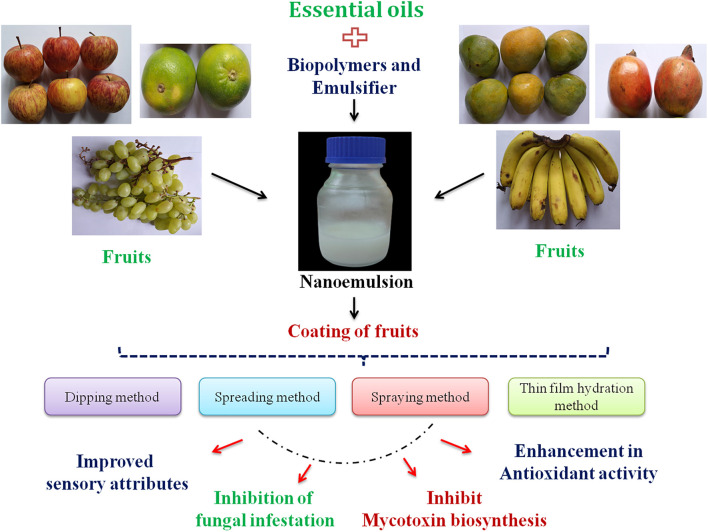
Essential oil nanoemulsion coating of fruits by different methods and their practical application for fruit quality maintenance.

After preparation of essential oil nanoemulsion-based coating dispersion, the fruits are well mixed with the solution to attain homogeneous coating around the surface. Four different procedures, *viz*., dipping, brushing, spraying, and film hydration methods, are applied for successful coating of fruits (Ju et al., 2019). In the case of the dipping technique, the fruits are directly immersed into the coating dispersion for 2–4 min followed by drying for a certain period of time (Chaudhary et al., 2020). This technique usually forms high-thickness coating, maintaining the viscosity, density, and surface tension of the coating solution. Tharanathan (2003) applied the foam method for fruit coating with uniform distribution and repeated rolling action. Mastromatteo et al. (2012) developed coating of carrot by sodium alginate and sodium hydroxide and extended the shelf life by 7 days. In the spreading process, the coating dispersion is directly brushed on the surface of the fruit to form a uniform layer (Chaudhary et al., 2020). The spraying technique includes low-viscosity homogeneous coating of fruits by using high pressure. In the case of the film hydration method, essential oil-loaded emulsion is mixed with organic solvent followed by evaporation of the solvent through a rotatory evaporator and finally hydration by the addition of an appropriate buffer. This is a basic method of coating with high encapsulation efficiency. Alikhani-Koupaei (2015) demonstrated lemon essential oil-loaded liposome coating on spinach to maintain nutritional qualities. Figure 1 presents essential oil nanoemulsion coating of fruits by different methods and their practical application for fruit quality maintenance.

## Essential Oil-Based Edible Coating on Fruits Against Fungal Infestation and Mycotoxin Contamination

Judgment of fruit quality has been done through freshness as well as external appearance at the time of purchase. Minimal processing can alter the integrity of fruits, resulting in a negative impact on fruit quality like browning, breakdown of texture, and off-flavor that may lead to fungal occurrence, compromising the safety of the fruits. Emulsion-based edible coating has shown promising effects to maintain the physicochemical qualities and nutritional behaviors of fruits during postharvest storage. Table 1 presents the essential oil-based nanoemulsion edible coating of different fruits by using different coating wall polymers and their effects against microbial contamination.

**TABLE 1:** Essential oil-based nanoemulsion edible coating of different fruits by using different coating wall polymer and their effects against microbial contamination.

**Type of coating**	**Essential oils**	**Fruits**	**Application methods**	**Major findings**	**References**
Chitosan	Mentha piperita and Mentha villosa	Grape	Dipping	Significant efficacy against phytopathogenic fungi	Guerra et al., 2016
	Lemongrass essential oil			Effectively inhibit growth of yeast	Oh et al., 2017
	Bergamot essential oil			Maintained the quality of grapes	Sánchez-González et al., 2011
Hsian-tsao leaf gum and tapioca starch	Cinnamon essential oil	Apple	Dipping and spraying	Delay in browning	Pan et al., 2013
Gellan and alginate	Rosemary and cinnamon essential oil			Effective inhibition of microbial growth	Rojas-Argudo et al., 2009; Chiabrando and Giacalone, 2015
Sodium alginate and Tween-80	Lemongrass			Effective inhibition of microbial growth	Salvia-Trujillo et al., 2015
Carrageenan and alginate	Lemongrass essential oil	Pine apple	Spreading	Active against microbial deterioration and also maintain sensory attributes	Azarakhsh et al., 2014
Flaxseed gum	Lemongrass essential oil	Pomegranate aril	Dipping	Inhibition of yeast and mold growth	Yousuf and Srivastava, 2017
Starch	Nigella sativa			Reduced loss of anthocyanin and vitamins	Oz and Ulukanli, 2012
Basil seed gum	Origanum vulgare	Apricot	Spraying	Inhibition of yeast growth and facilitation of shelf life extension	Hashemi et al., 2017
Chitosan	Lemon essential oil	Strawberry	Dipping	Reduction in respiration rate during storage and inhibition of fungal growth	Perdones et al., 2012
Carnauba wax and Tween-80	Lemongrass	Plums	Spreading	Inhibition of microbial growth	Kim et al., 2013
Polysorbate 20/sodium lauryl sulfate	Thymol	Blueberry	Dipping	Inhibition of foodborne biofilm	Li et al., 2017
Chitosan and cassava starch	Myrcia ovata	Mangaba	Dipping, spraying, and spreading	Inhibition of foodborne microbe during storage	Frazão et al., 2017
Alginate and pectin	Lemon essential oil	Raspberries	–	Coating maintained raspberry color and reduced weight loss	Guerreiro et al., 2015
Rhamnolipid and sodium carbonate	Cinnamon essential oil	Cherry	Dipping	Inhibit growth of microorganism and lipid peroxidation	Xu, 2016

Pear (*Pyrus malus* L.) is an economically important fruit in temperate zones of the world (Silva et al., 2014). The fruits maintain acid balance in the human body and are a prime source of pectin (Dave et al., 2017). During the postharvest period, rapid softening and senescence of the outer tissue with resultant fungal proliferation pose a serious problem for marketing. Gago et al. (2020) investigated the effect of alginate-based (2% w/w) nanoemulsion incorporated with lemongrass essential oil (1.25% w/w) for the preservation of Rocha pear (at 22°C and 70% relative humidity for 7 days), maintaining the fruit’s color, soluble solid contents, titrable acidity, and firmness. More importantly, the nanoemulsion retarded the ripening process of fruits and exhibited a positive impact on fruit qualities.

Papaya (*Carica papaya*) is a common fruit and has been reported to possess high amounts of carotenoids, calcium, and ascorbic acid (Zillo et al., 2018). Papaya has broad nutritional and health beneficial effects, including prevention of arteriosclerosis, cancer, and heart problems (Ali et al., 2011). Due to its climacteric nature, the fruit has a very short postharvest life and is generally susceptible to fungal infestation, increased softening of pulp, and excessive accumulation of sugar and water (González-Aguilar et al., 2009). Generally, papaya is consumed after peeling, seeding, and cutting, which induce the respiration rate, leading to biochemical changes and desiccation causing effective fungal spoilage. Essential oil nanoemulsion-based edible coating provides a primary packaging to papaya which effectively improves its internal atmosphere and control the moisture and gas content of the fruit. Tabassum and Khan (2020) studied the impact of alginate-mediated (2% w/v) edible coating incorporating *Thymus vulgaris* and *Origanum vulgare* essential oil (0.5, 1.0, and 2.0%) nanoemulsion in papaya on preservation against molds and yeast spoilage over a period of 12 days at 4°C. Yeast and mold counts of uncoated fruit samples reached up to 5.90–8.40 CFU/g, which was much higher than the consumable limit.

Strawberries are one of the economically important fruits and recognized worldwide due to its desirable flavor and taste, with rich bioactive components such as vitamin E, vitamin C, anthocyanin, and β-carotene providing a number of health benefits (Van De Velde et al., 2013). However, the perishability and high respiration rate lead to rapid senescence and reduce the shelf life of fruits. Moreover, the mechanical injuries cause infestation of several mold species, culminating into effective changes in firmness, color, and quality of fruits (Treviño-Garza et al., 2015). Chu et al. (2020) synthesized the pullulan coating (2% w/v) by incorporation of cinnamon essential oil nanoemulsion for strawberry fruits and studied its effect against mold and yeast contamination. The results indicated a reduction in decay percentage as compared to control during 6 days of storage period at 20 ± 2°C. In the case of control and pure pullulan-coated fruits, decay has started after 2 days, while the decay percentage reached up to 65% within 6 days. The essential oil-based pullulan coating of fruits reduced decay percentage by 30% after 6 days, suggesting the excellent antifungal activity of the essential oil nanoemulsion due to cinnamaldehyde as the major component, and inhibited fungal growth by membrane disintegration. The pullulan-incorporated cinnamon essential oil nanoemulsion coating retarded the fungal growth from 10^4^ to 1.958 CFU/g, which has been associated with the nanometric size of essential oil droplets with increased surface area and better membrane accessibility. Similar to the previous investigation, Robledo et al. (2018) demonstrated the effectiveness of thymol nanoemulsion-based (1:5 w/v) edible coating of strawberry fruits by using chitosan and quinoa protein as a wall matrix. During the whole course of storage periods, thymol nanoemulsion-mediated packaging maintained a lower yeast and mold count as compared to control (uncoated fruits) up to 10 days of storage. Fruits only coated with quinoa protein and chitosan showed lower effectiveness, which could have been due to the interaction of chitosan and quinoa protein. The greater mold and yeast inhibitory effects of thymol nanoemulsion have been associated with the controlled release of thymol from the biopolymer. Moreover, thymol nanoemulsion coating reduced the fungal decay of fruits by 41.7% after 16 days. The author demonstrated that chitosan and quinoa protein are the best coating material for synthesis of edible coating in strawberry fruits, with great reduction capability of fungal contamination.

Blueberries are considered as “super food” due to the presence of different beneficial and bioactive compounds facilitating healthy growth and development. The fruits are highly susceptible to fungal association, *viz.*, species of *Penicillium*, *Alternaria*, *Cladosporium*, *Botrytis*, and *Fusarium* and their associated mycotoxin contamination (Stinson et al., 1980; Tournas and Katsoudas, 2005; Munitz et al., 2013). In a study of Umagiliyage et al. (2017), D-limonene (10–50 μM) encapsulated into liposome showed prominent inhibition of fungal decay (7.5%) over a period of 63 days. Moreover, the phospholipid layer around D-limonene in the liposome system restricted the degradation and facilitated controlled volatilization with targeted delivery and better action.

*Citrus* fruits are severely infested by different storage fungi such as *P. expansum*, *Penicillium digitatum*, and *A. flavus*, leading to 90% of postharvest loss. Edible coating of essential oils and their component-assisted nanoemulsion with high water solubility developed a potential strategy for inhibition of fungal proliferation by preventing degradation of active constituents along with improved bioavailability. Yang et al. (2021) reported the antifungal effect of stable nanoemulsion containing carvacrol, eugenol, and cinnamaldehyde (0.0078–0.5 mg/ml) against *P. digitatum* with a resultant increase in fruit shelf life. Results represented that the fungal decay rate was reduced to 4.1% after nanoemulsion coating of fruits as compared to control groups (7.4%) after 60 days of storage. Nanoemulsion coating also reduced the weight loss from 4.12 to 3.14%, which has been associated with the nano-range size with greater surface-to-volume ratio and better fungal inhibitory efficacy.

Grapes are rich source of phenolic compounds possessing antioxidant activities and play an active role against cardiovascular and neurodegenerative diseases (Sun et al., 2010; Mattioli et al., 2020). During postharvest storage, table grapes are infested by a number of mold species such as *A. niger*, *Botrytis cinerea*, *Rhizopus stolonifer*, and *P. expansum*, leading to loss in fruit quality. A recent investigation of Guerra et al. (2016) suggested the significant protection of table grapes against *B. cinerea*, *R. stolonifer*, and *P. expansum* (32–38% of fruit was infected) by chitosan-incorporated edible coating of *M. piperita* and *Mentha villosa* essential oils (1.25, 2.5, and 5.0 μl/ml).

## Antifungal and Antimycotoxigenic Mechanism of Action

As essential oils are mixtures of variable bioactive components, a number of target sites have been demonstrated for antifungal and antimycotoxigenic activity by different researchers. Moreover, hydrophobic essential oils easily traverse the lipid bilayer of the plasma membrane and affect the synthesis of ergosterol along with enhanced efflux of vital cellular ions, leading to disintegration of membrane stability and permeability. The lipophilic nature of essential oils also allows disruption of the cell wall compatibility by targeting polysaccharide back bones (Rammanee and Hongpattarakere, 2011). Improvement in antifungal activity of essential oils in a nanoemulsion system has been associated with increased solubility and changes in permeability of the plasma membrane by modification of the cellular pH and affected osmotic pressure (Chaudhari et al., 2021). Manso et al. (2013) reported the disruption in hyphal structure and conidial wall of *A. flavus* after fumigation with cinnamon essential oil (0.1 mg/ml). Abd-Elsalam and Khokhlov (2015) reported antifungal activity of nanoencapsulated eugenol (2%) against *Fusarium oxysporum* due to the inhibition of conidial germination. Long et al. (2020) investigated the effect of garlic essential oil nanoemulsion (3.7%) against the growth of *Penicillium italicum* due to the penetration of active components and interaction with fungal cellular enzyme, culminating in cell death. A little increment in extracellular conductivity after fumigation with garlic essential oil nanoemulsion has been linked with the inhibition of spore germination. A recent investigation of Das et al. (2021c) suggested dose-dependent retardation of cellular methylglyoxal by eugenol-loaded chitosan nanoemulsion (0.07 μl/ml), leading to the inhibition of cellular aflatoxin biosynthesis in *A. flavus*. Wan et al. (2019) reported a remarkable negative impact of peppermint (2.5–25 mg/g), thyme, cinnamon, clove, and lemongrass essential oil (2–10 mg/g) nanoemulsion on the growth of *Fusarium* sp. and production of deoxynivalenol, 15-acetyldeoxynivalenol, and 3-acetyldeoxynivalenol based on changes in fungal metabolic activities. Hu et al. (2017) demonstrated an antiaflatoxigenic mechanism of action of *Curcuma longa* essential oil due to the downregulation of five structural genes, *viz*., Afl O, Afl M, Afl D, Afl P, and Afl Q, in *A. flavus*. El Khoury et al. (2016) pointed out the inhibition of ochratoxin A biosynthesis by essential oils (1.0 and 5.0 μl/ml) due to downregulation of polyketide synthase, acpks, and acOTAnrps genes in fungal cells.

Recently, an *in silico* modeling study also revealed the binding interaction of essential oil components with mycotoxin-biosynthesizing proteins, demonstrating a new horizon for molecular target sites of action. The *in silico* interaction of α-pinene, apiol, elemicin, *p*-cymene, and fenchone with the ergosterol-biosynthesizing protein lanosterol-14α-demethylase and the aflatoxin-producing proteins polyketide synthase and Ver-1 suggested a molecular target site for antifungal and antiaflatoxigenic activities (Das et al., 2021d). Pani et al. (2016) suggested a better understanding of the structure–activity relationship of phenolic components isolated from higher plants with the trichothecene biosynthetic gene trichodiene synthase TR 15 by molecular docking for the inhibition of trichothecene production in *Fusarium culmorum*. *In silico* molecular interaction of phytochemicals, *viz*., hexanoic acid and quercetin, with seven different domains of polyketide synthase for the inhibition of aflatoxin biosynthesis in *A. flavus* has been recently investigated by Tiwari et al. (2019). Conclusively, better elucidation of complex molecular interactions of essential oil components and target proteins could provide a new basis for synthesizing novel green antifungal components. Table 2 represents the antifungal and antimycotoxigenic mechanisms of action of essential oil-loaded nanoemulsion.

**TABLE 2:** Antifungal and antimycotoxigenic mechanisms of action of essential oil/components-loaded nanoemulsion.

**Essential oils/components nanoemulsion**	**Active against**	**Mechanism of action**	**References**
Garlic oil	*Penicillium italicum*	Destruction of membrane permeability and stability	Long et al., 2020
Cinnamon oil	*Fusarium graminearum*	Loss of cytoplasmic constituents	Wu et al., 2019a
Cinnamon oil	*Rhizopus* sp.	Membrane disruption	Yousef et al., 2019
Myristica fragrans	*Aspergillus flavus*	Leakage of vital ions and UV-absorbing materials	Das et al., 2020a
Eugenol	*Fusarium oxysporum* f. sp. vasinfectum	Inhibition of conidial germination	Abd-Elsalam and Khokhlov, 2015
Eugenol, cinnamaldehyde, and carvacrol	*Penicillium digitatum*	Destruction of membrane permeability	Yang et al., 2021
Lemongrass and clove	*Fusarium oxysporum* f. sp. *lycopersici*	Destruction of membrane integrity	Sharma et al., 2018
Illicium verum	*Aspergillus flavus*	Leakage of Ca^2+^, K^+^, and Mg^2+^ ions	Dwivedy et al., 2018
Cleome viscosa	*Candida albicans*	Inhibition of cell wall biosynthesis	Krishnamoorthy et al., 2021
Citrus sinensis	*Saccharomyces cerevisiae*	Distortion in cellular morphology	Sugumar et al., 2016b
Zataria multiflora and Carum copticum	*Byssochlamys fulva*	Inhibition of mycelia growth	Sahraneshin Samani et al., 2019
Coriandrum sativum	*Aspergillus flavus* and *aflatoxin*	Disruption of membrane permeability and inhibition of methylglyoxal biosynthesis	Das et al., 2019
Origanum majorana	*Aspergillus flavus* and *aflatoxin*	Leakage of Ca^2+^, K^+^, and Mg^2+^ ions and inhibition of methylglyoxal biosynthesis	Chaudhari et al., 2020
Anethum graveolens	*Aspergillus flavus* and *aflatoxin*	Inhibition of spore germination	Das et al., 2021b
Clove, black seed, lemon and orange essential oil	*Alternaria tenuissima, Galactomyces candidum*, and *Fusarium solani*	Inhibition of conidial sporulation	Mossa et al., 2021
Cinnamomum zeylanicum	*Fusarium graminearum*	Inhibition of conidial germination	Wu et al., 2019b
Citrus reticulata	*Aspergillus niger* and *Zygosaccharomyces rouxii*	Inhibition of mycelial growth	Mahdi et al., 2021
Orange essential oil	*Aspergillus flavus*	Inhibition of mycelial growth	Kringel et al., 2021
Zataria multiflora and Bunium persicum	Yeasts and molds	Slowdown of spore germination, radial growth, and germ tube elongation	Keykhosravy et al., 2020
Limonin	*Penicillium italicum*	Inhibition of ergosterol biosynthesis	Li et al., 2021
Litsea cubeba	*Penicillium italicum* and *Penicillium digitatum*	Inhibition of mycelial growth	Wang et al., 2020

## Impact of Edible Coating on Antioxidants of Fruits

Fruits are good sources of antioxidant due to the presence of higher amounts of phenolic constituents and anthocyanins. Phenolic components provide the sensory and nutritional properties of many fruits. During the storage period, the oxidation of phenolic components by polyphenol oxidase is evidenced by dark-colored pigments, and the overall antioxidant activity is reduced (Orak, 2007). Moreover, the subsequent degradation and decline in phenolic content are also associated with cell structure breakdown and progressive fruit senescence. Edible coating of essential oils has the potentiality to preserve flavonoids, phenolics, carotenoids, lycopenes, and glucosinolates by reducing fruit deterioration (Maringgal et al., 2020). Essential oils facilitate the reduction of gas exchange and inhibition of carbohydrate decomposition by retarding the respiration process. Essential oil-incorporated nanoemulsion of edible coating is an innovative strategy for controlled delivery by immobilization of droplets, allowing stability against aggregation and increased bioavailability on the surface of the fruits and significantly maintaining the antioxidant activity. The particle size of essential oil-based nanoemulsion has been found as an important index for regular distribution and homogeneous edible coating. Sometimes, the phenolic content of essential oil may induce the overall phenol level in fruits. Retention of phenolic components in fruits by essential oil nanoemulsion coating may also depend on the type and concentration of essential oils in nanoformulation. Additionally, an increase in cellular phenylalanine ammonia lyase activity may also induce the total phenolic content of fruits (Guerreiro et al., 2017).

Moreover, during postharvest storage, the toxic free radical molecules are accumulated, causing maximum loss of nutritional quality of fruits, whereas catalase, peroxidase, superoxide dismutase, and ascorbate peroxidase are important antioxidant defense enzymes that can easily scavenge the ROS and make the fruits fresh with good antioxidant ability. Yang et al. (2021) reported that carvacrol, eugenol, and cinnamaldehyde nanoemulsion-based (0.0078–0.5 mg/ml) coating of citrus fruits was effective in increasing catalase, peroxidase, and superoxide dismutase by 22.29, 51.49, and 18.12%, respectively, after a period of 40 days of storage.

Antioxidant activity in fruits is easily measured through inhibition of the 2,2-diphenyl-1-picryl-hydrazyl-hydrate (DPPH) radical. Edible coating may act as an effective barrier against oxygen uptake and significantly reduce antioxidative agents (Bonilla et al., 2012). Essential oil-based nanoemulsion also provide pronounced effects on antioxidant potency of fruits due to increased antioxidant compounds with greater ROS scavenging ability. Cenobio-Galindo et al. (2019) studied the effect of *Citrus sinensis* essential oil coating on total phenolics, flavonoids, and antioxidant activity of avocado fruits. They designed the coating experiments on the basis of four different treatments, *viz*., concentrated nanoemulsion (CN), 50% diluted nanoemulsion (N50), 25% diluted nanoemulsion (N25), and the control set (C). Among different treatment sets, the highest phenolic (240.15 and 214.29 mg gallic acid equivalent/100 g) and flavonoid contents (47.77 and 48.18 mg quercetin equivalent/100 g) were recognized for N50 and N25 after 60 days of storage. Increment in DPPH and 2,2′-azino-bis (3-ethylbenzothiazoline-6-sulfonic acid) (ABTS) antioxidant activity of avocado mesocarp was also exhibited for N50 and N25 treatments as compared to the control. Authors demonstrated the antioxidant activity maintenance of fruits due to controlled delivery of *C. sinensis* essential oil with greater surface-to-volume ratio in the nanoemulsion system.

## Impact of Edible Coating on Physicochemical Parameters and Sensory Properties of Fruits

Encapsulation of essential oil and their controlled as well as targeted delivery can maintain the chemical attributes of fruits during storage. The edible coating has a positive impact on fruit freshness without affecting total soluble solids (TSS), titrable acidity, and pH. Chu et al. (2020) represented significant maintenance of TSS by cinnamon essential oil-loaded pullulan coating in strawberry during storage. The stable nature of TSS after essential oil-based edible coating has been associated with considerable retardation of conversion of reducing sugar in strawberry. Moreover, the pullulan–cinnamon essential oil nanoemulsion slowed down the respiration rate and metabolic activities possibly related with the interactions of cinnamon essential oil and the plasma membrane of strawberry. Sometimes, the edible coating of fruits also induces the TSS value, which may be due to the water loss during the storage periods and rapid ripening process. Therefore, the decline or stable nature of TSS value in fruits after nanoemulsion coating suggests effective barrier properties against water loss. Decreasing firmness and consistent weight loss are major problems for the preservation of tomato fruits. The investigation of Athayde et al. (2016) suggested that weight loss of tomato by treatment with chitosan-incorporated *Cymbopogon citratus* essential oil coating was lower as compared to uncoated tomato fruits. Moreover, the coating of tomato fruits also facilitated increment of ascorbic acid, pH, soluble solid, and titrable acidity, leading to better acceptance of market value.

In spite of better preservation potentiality, the effect of essential oil-based edible coating on sensory properties of fruits is one of the major aspects that deal with the decision of consumers during purchase. As essential oils contain strong aroma, any changes in flavor and taste of the fruits may obstruct its quality attributes resulting from interaction with components in fruit tissue. The recent investigation of Mohammadi et al. (2021) reported *Aloe vera* gel coating enriched with *Ocimum basillicum* essential oil to conserve the sensory properties of strawberry fruits. Treatment of fruits with *A. vera* gel alone and two different concentrations, *viz.*, 500 and 1,000 μl/L, of *O. basillicum* essential oil displayed the highest scores as compared to distilled water-treated and control fruit samples in the storage conditions. Saki et al. (2019) also revealed the decreasing sensory scores of fig fruits over 20 days of storage, whereas fig fruits fumigated with 200 mg/L thymol incorporated into 0.5% chitosan showed maximum overall acceptability (score 3.66) as compared to separate fumigation of 200 mg/L thymol (score 1.66) and 0.5% chitosan (score 2.00). They also demonstrated the improvement in sensory attributes due to lower water loss from the fruit surfaces and maximum balance between acids and sugars of fruits.

## Conclusion and Future Direction of Research

The safety and quality of fruits are of increasing concern because contaminated fruits can act as a vehicle for transmission of hazardous diseases. Essential oils have been utilized as a green alternative of synthetic preservatives with proven antifungal and antimycotoxigenic activities. Encapsulation of essential oils into biodegradable and biocompatible polymers forming a nanoemulsion system improves their solubility, stability, and efficacy and minimizes fungal as well as mycotoxin contamination. Moreover, nanoemulsion-mediated edible coating of fruits further ensures the maintenance of antioxidant activities, physicochemical properties, and organoleptic attributes with resultant enhancement in shelf life.

Encapsulated essential oils in a nanoemulsion system offer a number of benefits for fruit preservation; however, several challenges have to be focused during commercial exploitation. For instance, the combinatorial action of essential oil and components in a synergistic nanoemulsion system, judicious selection of essential oils, and more importantly, the safety profile of essential oil nanoemulsion should be worked out for large-scale practical recommendation as a novel shelf-life enhancer for stored fruits.

## Author Contributions

SD gave the idea and concept of the review article and wrote the original manuscript. AG and AM performed the review of literature. All authors read and approved the manuscript.

## Conflict of Interest

The authors declare that the research was conducted in the absence of any commercial or financial relationships that could be construed as a potential conflict of interest.

## Publisher’s Note

All claims expressed in this article are solely those of the authors and do not necessarily represent those of their affiliated organizations, or those of the publisher, the editors and the reviewers. Any product that may be evaluated in this article, or claim that may be made by its manufacturer, is not guaranteed or endorsed by the publisher.
